# Supplementary material for: Neuroreceptor Inhibition by Clozapine Triggers Mitohormesis and Metabolic Reprogramming in Human Blood Cells
Source: Cells. 2024 Apr 29;13(9):762. doi: 10.3390/cells13090762 (PMC11083702; doi:10.3390/cells13090762)
Supplement: Supplementary file 1 [file cells-13-00762-s001.zip › cells-2982433-supplementary.pdf]

Table S1 suppl. Primer sequences used for RT-PCR in Fig. 2C

mRNA	Acc.Nr	forward primer	reverse primer	length
HIF-1a	NM_001530	5`-GCTGGCCCCAGCCGCTGGAG-3`	5`-GAGTGCAGGGTCAGCACTAC-3`	214 bp
ß-actin	NM_001101	5`-GACTATGACTTAGTTGCGTTA-3`	5`-GTTGAACTCTCTACATACTTCCG-3`	459 bp
GRP78	NM_005347	5`-GTTCTTGCCGTTCAAGGTGG-3`	5`-TGGTACAGTAACAACTGCATG-3`	181 bp
SIRT3	NM_001017524	5`-CCTGTGACTTTGCGCCTTA-3`	5`- CCACACATAGCCACAGAAACA-3`	464 bp
SIRT6	NM_001193285	5`-TGTTACTTGTTTCTGTCCCCG-3`	5`-AAGGCAGTGCAAGCCTCTAC-3`	362 bp
ATP-IF1	NM_016311	5`-CGTAACGAGAGACTGCTTGCT-3`	5`-CACAGAAGTGGGCAATGACA-3`	426 bp
SOD2	NM_000636.2	5`-AACGCGCAGATCATGCAGCTGC-3`	5`-CCCAGTTGATTACATTCCAA-3`	505 bp
PDK3	NM_005391	5`-TTAATAAGTCCGCATGGCGC-3`	5`-TGAAGCATCCCTGGGTTCAC-3`	86 bp
Catalase	NM_001752	5`-TTTGGCTACTTTGAGGTCAC-3`	5`-TCCCCATTTGCATTAACCAG-3`	440 bp

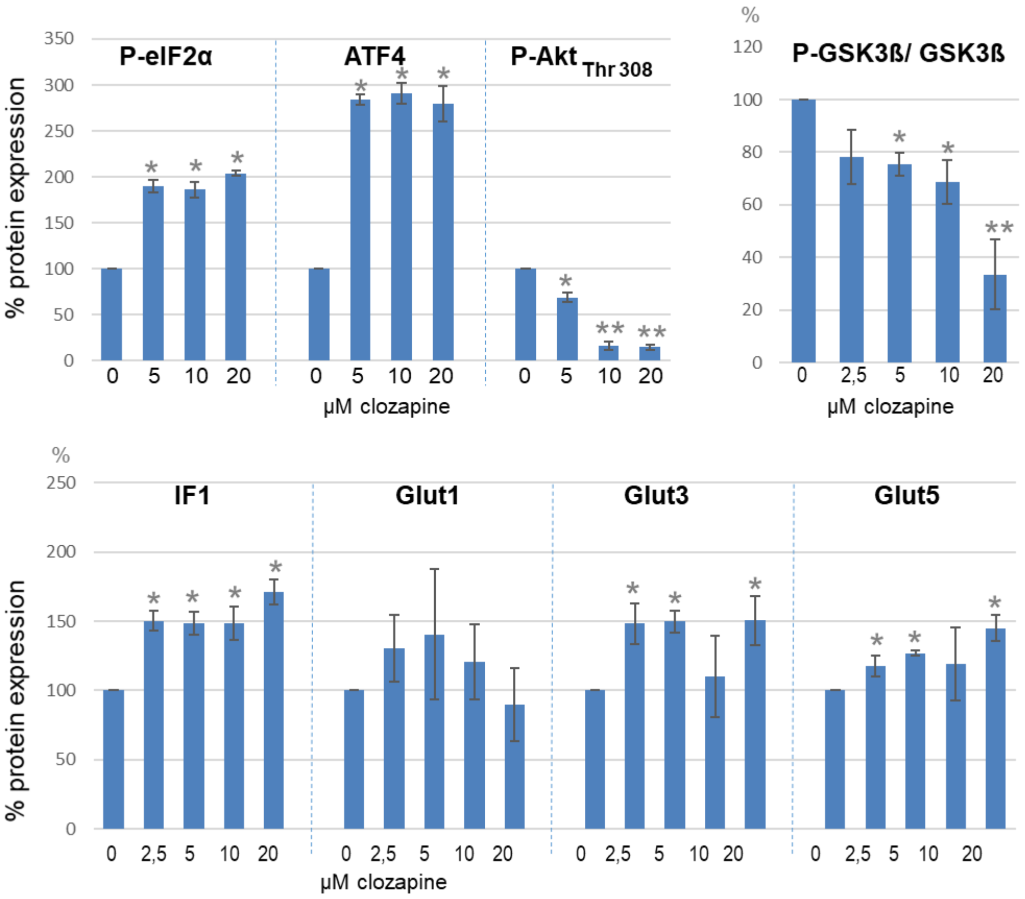

Figure S1 suppl. Densitometric evaluation of Western Blots

Western blotting analysis of GLUT1, Glut3, GLUT5, IF1, P-eIF2α, ATF4, P-Akt thr308 contents, and the P-GSK3ß/ GSK3ß ratio of HL60 cells obtained from control group (0µM clozapine) and clozapine-treated groups. Graphics show the relative density of protein levels with the control values set as 100%. Values represent mean ± SEM. Statistical analysis was calculated by Kruskal-Wallis tests. Asterisks denote significant difference (* $p < 0.05$; ** $p < 0.005$) from values for control group.

Figure S2 suppl. Chemiluminescence detection of the membranes incubated with the primary antibodies against GSK3ß, P-GSK3ß, Glut1, Glut 3, Glut5, IF1, P-Akt Thr308 and the ER stress proteins ATF4 and eIF2α and their respective secondary antibodies, (HRP-conjugated).

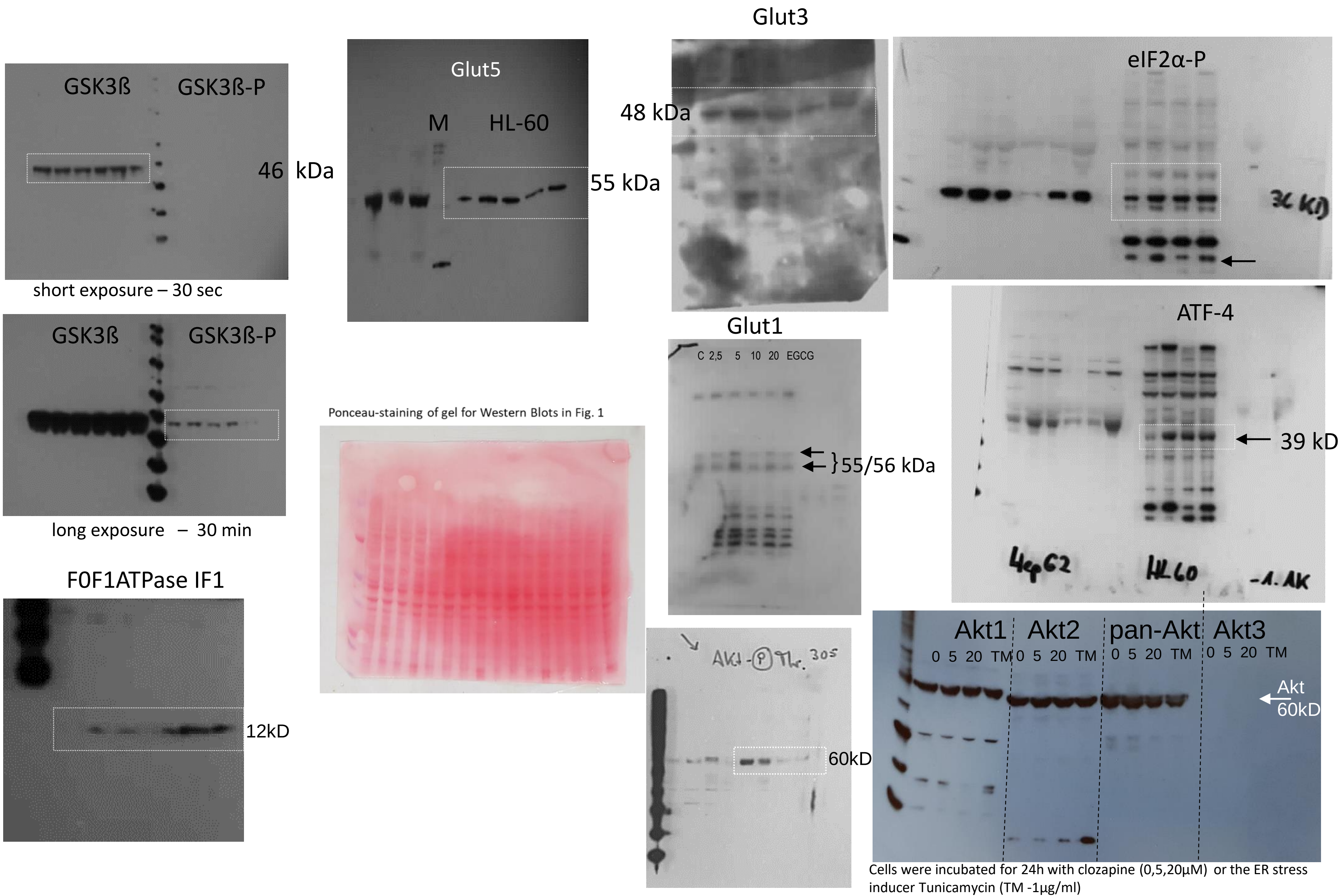

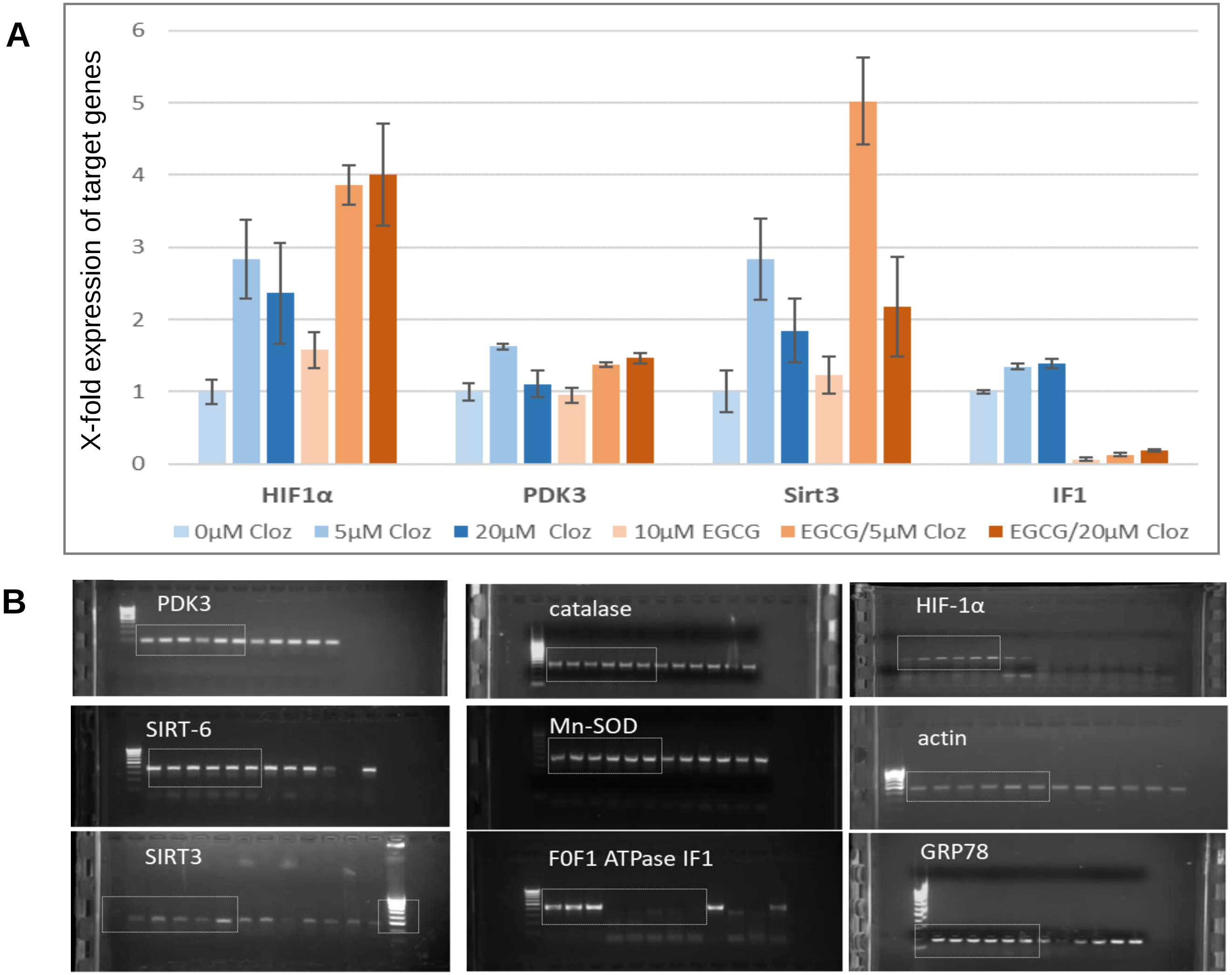

Figure S3 suppl. A) Presentation of the quantitative results of the PCR analyses shown in Fig. 2C. The target gene/actin ratio for the untreated cells was set as 1. Further statistical analysis by Kruskal-Wallis tests revealed that 4 of the 8 target genes were significantly affected by clozapine dosage (HIF1α: $H(3) = 9.154$, $p = 0.027$; PDK3: $H(3) = 9.051$, $p = 0.029$; Sirt3: $H(3) = 9.154$, $p = 0.027$ and IF1: $(H3) = 9.974$, $p = 0.019$).
B) Gel electrophoresis of the RT-PCR products presented in Fig. 2C. Photographs show the complete agarose gels from which the area of the corresponding bands was cut off.

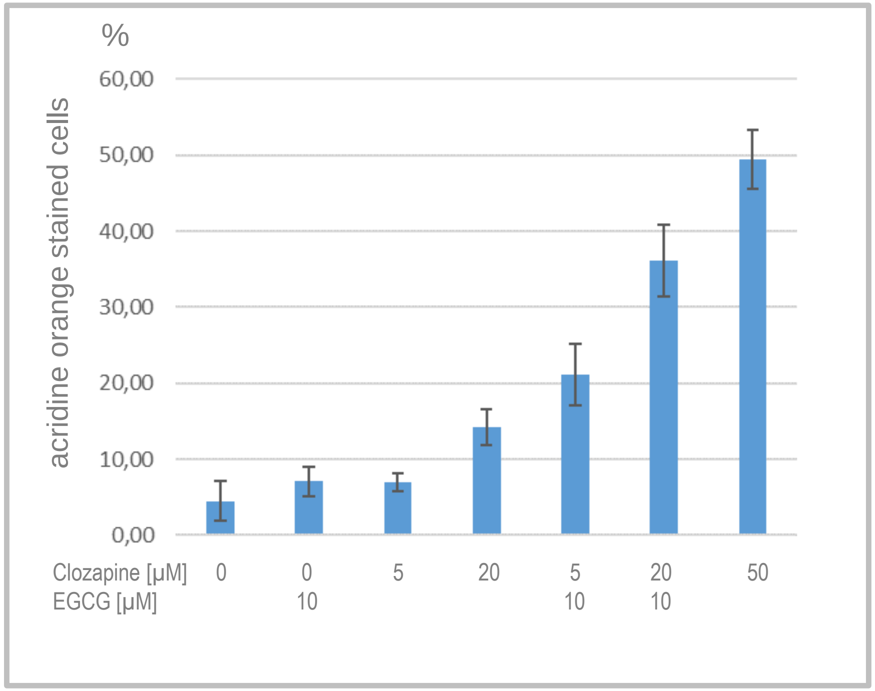

Figure S4 suppl. Presentation of the quantitative results of the acridine orange staining shown in Fig. 2F.

Statistical analysis by the Kruskal-Wallis test revealed that acridine orange staining was significantly affected by clozapine dosage, $H(6) = 18.900$, $p = 0.004$).

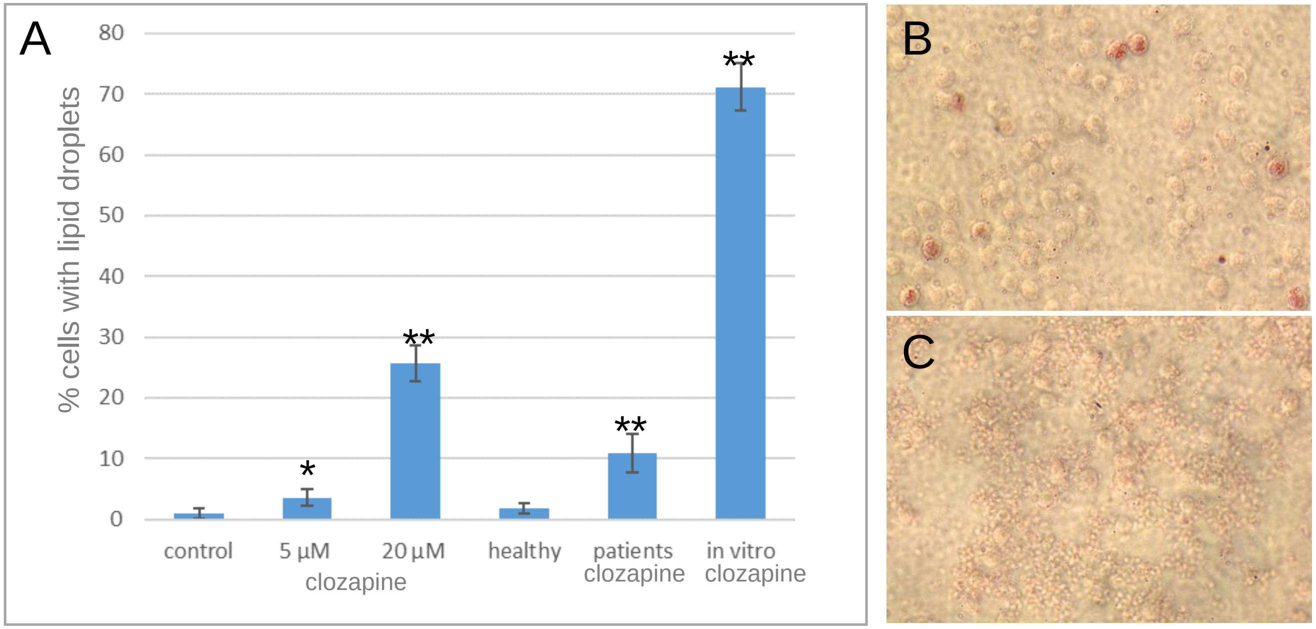

Figure S5 suppl. A) Presentation of the quantitative results of the oilred staining shown in Fig. 4C. Statistical analysis (nonparametric Kruskal-Wallis test) revealed that the number of lipid droplet containing HL60 cells was significantly affected by clozapine dosage ($H(6) = 18.900$, $p = 0.004$) as well as blood cells from clozapine treated patients ($H(2) = 9.000$, $p = 0.003$). *In vitro* culture of blood cells with clozapine [20µM] for 8h led to a significant increase of lipid droplets in about 70% of cells. B) Oilred stained only neutrophils from patients under clozapine therapy but not their lymphocytes (C).

Analysis of Intracellular Generation of Reactive Oxygen Species
Intracellular generation of superoxide anions was determined by monitoring the oxidation of hydroethidine (Sigma, Deisenhofen, Germany) to ethidium bromide, as previously described in [42]. About 2 × 10^6 leukocytes were incubated in RPMI/10% FCS in the presence of 2 μM dihydroethidine without further stimulation for 1 hour at 37°C prior to microscopic evaluation. Red fluorescence of the cells revealed the production of superoxide anion radicals measured by metabolic rate of hydroethidine oxidation.

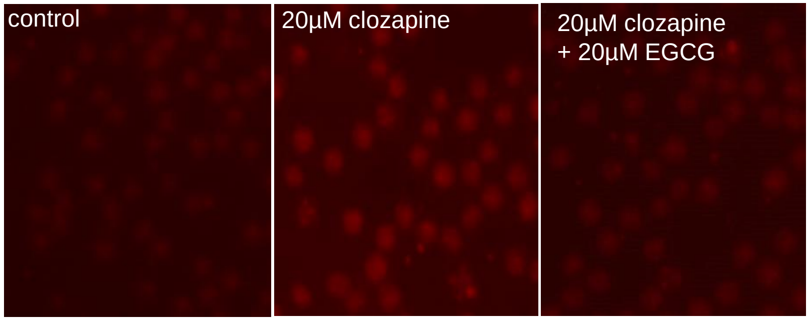

Figure S6 suppl. HL60 cells treated for 24h with 20µM clozapine showed red fluorescence, indicating superoxide radical production. Cotreatment with EGCG inhibited ROS production.
